# Brain-Derived Neurotrophic Factor Signaling in the Pathophysiology of Alzheimer’s Disease: Beneficial Effects of Flavonoids for Neuroprotection

**DOI:** 10.3390/ijms22115719

**Published:** 2021-05-27

**Authors:** Tadahiro Numakawa, Haruki Odaka

**Affiliations:** 1 Department of Cell Modulation, Institute of Molecular Embryology and Genetics, Kumamoto University, Kumamoto 860-0811, Japan; numakawa.yyrmk@gmail.com; 2 Cellular and Molecular Biotechnology Research Institute, National Institute of Advanced Industrial Science and Technology (AIST), Central 6, Ibaraki 305-8566, Japan

**Keywords:** brain-derived neurotrophic factor, Alzheimer’s disease, intracellular signaling, flavonoids

## Abstract

The function of the brain-derived neurotrophic factor (BDNF) via activation through its high-affinity receptor Tropomyosin receptor kinase B (TrkB) has a pivotal role in cell differentiation, cell survival, synaptic plasticity, and both embryonic and adult neurogenesis in central nervous system neurons. A number of studies have demonstrated the possible involvement of altered expression and action of the BDNF/TrkB signaling in the pathogenesis of neurodegenerative diseases, including Alzheimer’s disease (AD). In this review, we introduce an essential role of the BDNF and its downstream signaling in neural function. We also review the current evidence on the deregulated the BDNF signaling in the pathophysiology of AD at gene, mRNA, and protein levels. Further, we discuss a potential usefulness of small compounds, including flavonoids, which can stimulate BDNF-related signaling as a BDNF-targeting therapy.

## 1. Introduction

It is well-known that the function of the brain-derived neurotrophic factor (BDNF) via stimulating its high-affinity receptor tropomyosin receptor kinase B (TrkB) contributes to cell differentiation, cell survival, synaptic plasticity, and both embryonic and adult neurogenesis in central nervous system (CNS) neurons. In addition to the contribution of the BDNF to physiological neural functions [1], studies have demonstrated possible involvement of altered expression and action of the BDNF in the pathogenesis of neurodegenerative diseases, including Alzheimer’s disease (AD), because an impaired neurogenesis during aging has been considered one of causes for the onset of brain diseases [2]. Postmortem study showed that hippocampal and cortical expression levels of the BDNF were significantly decreased [3,4,5]. The in vitro study also showed downregulation of the *BDNF* transcript after exposure to oligomeric amyloid beta (Aβ), which is considered as one of the risk factors in the pathogenesis of AD [6]. Rohe et al. reported that the BDNF upregulated sorting protein-related receptor with A-type repeats (SOLRA) expression via stimulating the extracellular signal-regulated kinase (ERK) signaling, and decreased amyloidogenesis in cultured cortical neurons [7]. Furthermore, it was demonstrated that marked dephosphorylation of tau protein occurred after BDNF application in differentiated P19 mouse embryonic carcinoma cells through the phosphatidylinositol-3 kinase (PI3K)/Akt signaling pathways [8]. It is possible that understanding the details of the behavior of the BDNF/TrkB system, which triggers several intracellular signaling including ERK and PI3K/Akt cascades, is essential to develop effective therapy for AD treatment.

Recently, a high association of pro-neurotrophins (including proBDNF, an immature BDNF form before processing) with the p75/sortilin receptor was focused. Using primary culture of hippocampal neurons, the cell-death promotion via p75/sortilin receptor-mediated signaling when exposed to cerebrospinal fluid (CSF) obtained from AD patients was reported [9]. The p75 receptor was initially recognized as a low-affinity common receptor for neurotrophin members. However, not only BDNF/TrkB-mediated neuronal events, but also action of the proBDNF/p75 system (which is highly interactive and negatively regulates neural aspects) should be also considered as a major contributor in neurodegenerative diseases. Therefore, to achieve selective activation of TrkB-mediated signaling, agonists for TrkB are useful targets in the amelioration of neuronal dysfunction. Especially, 7,8-dihydroxyflavone (7,8-DHF), one of the flavonoids, was used as an ideal agonist for the TrkB receptor to achieve neuroregeneration [10] because of a poor blood–brain barrier (BBB) penetration of the BDNF. 

In this review, we show the recent studies on possible contribution of BDNF-mediating intracellular signaling in neural function, and discuss finding concerning relationship between altered synaptic plasticity regulated by the BDNF and neurodegenerative diseases including AD.

## 2. The BDNF and Its Intracellular Signaling

### 2.1. The BDNF/TrkB Signaling-Mediated Pathways

**Figure 1:**
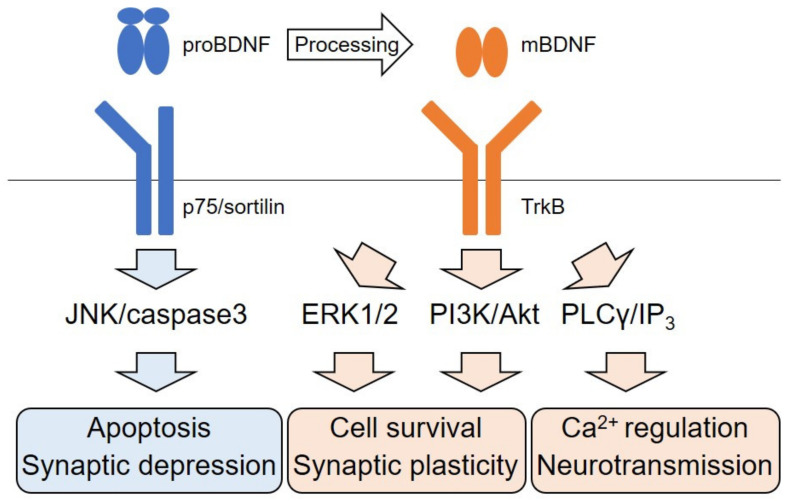
Schematic illustration of the BDNF signaling. The BDNF is initially translated as a precursor (proBDNF). proBDNF preferably binds with the p75-sortilin receptor complex and activates the JNK/caspase3 pathways which cause neural apoptosis and synaptic depression. proBDNF is proteolytically processed to a mature form (mBDNF). Binding of TrkB with mBDNF leads to activation of ERK1/2 and PI3K/Akt pathways to maintain cell survival and synaptic plasticity. mBDNF/TrkB also regulates Ca^2+^ homeostasis and neurotransmission via the PLCγ/IP_3_ pathway.

The BDNF, which exerts a critical role in neuronal differentiation, survival and synaptic function, belongs to neurotrophin family consisting of nerve growth factor (NGF), BDNF, neurotrophin-3 (NT-3), and NT-4 [11,12,13]. The expression of the BDNF and its high-affinity receptor, TrkB, are observed in the CNS expensively, although low-affinity p75 receptor, which is the common receptor for all neurotrophin members, also has a role in neuronal survival and synaptic function [13]. Stimulated TrkB after binding to the BDNF mainly activates three intracellular signaling pathways including ERK-, PI3K/Akt-, and phospholipase Cγ (PLCγ)- signaling (Figure 1) [14,15,16]. The ERK cascade is well studied, and research generally focuses on its role in neuronal differentiation and synaptic function [17,18]. Adequate activation of the ERK is required for upregulation of pre- and post-synaptic protein expression by the BDNF [19]. Remarkably, many studies have demonstrated the action of natural compounds for TrkB stimulation in the CNS. 7,8-DHF, one of the well-studied flavonoids, has survival promotion effect against traumatic brain injury [10]. Interestingly, studies have also demonstrated the beneficial effects of flavonoids in brain function via stimulating of cAMP response element binding protein (CREB), which is critical transcriptional molecule for expression of the BDNF and ERK activation [16]. Tea polyphenols (TP) also have survival-promoting effects through the TrkB/Akt signaling [20]. Yang et al. reported that rat hippocampal neurons exhibited neurite collapse and apoptosis in addition to reduced levels of proBDNF and downregulated TrkB/Akt signaling when exposing to staurosporine, a prototypical alkaloid. In contrast, the activation of ERK1/2 and caspase-3 were promoted after exposure to the alkaloid. Importantly, TP rescued the downregulation of proBDNF and TrkB/Akt signaling and protected hippocampal neurons against staurosporine-induced cytotoxicity. Recently, upregulation of the BDNF, TrkB, activated PI3K/Akt, and Bcl-2 (cell-survival-promoting molecule) by oral administration with Leonuri Herba Total Alkali (LHA) was reported [21]. Using in vivo rat cerebral ischemia model, a neuroprotective effect of the pre-administration with LHA was observed, suggesting that PI3K/Akt signaling is important for neuroprotection in cerebral ischemia. Ma et al. have reported a downregulation of the BDNF and TrkB/Akt/Fyn signaling, and an impairment in spatial memory caused by mangan (Mn) treatment [22]. Using in vivo and in vitro systems, they demonstrated that increased α-synuclein protein caused by Mn exposure contributed to reduced BDNF protein and inhibition of TrkB/Akt/Fyn signaling through interacting α-synuclein with TrkB, and resulting in repressed BDNF/TrkB signaling and impaired synaptic function.

### 2.2. Dichotomic Effects of ERK Signaling Activation on Neuronal Survival

Evidence has demonstrated an action of ERK signaling as cell-death promoter. We previously reported that overactivation of ERK signaling is involved in the cell death induction of cortical neurons under oxidative stress [23]. Huang et al. recently showed the contribution of ERK signaling in cell death caused by exposure to 4-methyl-2,4-bis(4-hydroxyphenyl)pent-1-ene (MBP), which is generated in the mammalian liver after exposure to bisphenol A, a harmful pollutant. In Neuro-2a cells, MBP exposure significantly induced overactivation of ERK and decreased Akt phosphorylation. Importantly, pretreatment with PD98059, an inhibitor for ERK1/2, and the overexpression of activation of Akt protected Neuro-2a cells against MBP-induced apoptotic events [24]. Although the difference between the BDNF-dependent ERK signaling and activation of this kinase under toxic environment is not clear, ERK signaling exerts a variety of roles in neuronal events as opposed to Akt signaling, which is mainly associated with neuronal survival.

### 2.3. Neurotransmitter Release Through the TrkB/PLCγ Pathway

It is well-known that the BDNF regulates intracellular Ca^2+^ dynamics via activation of the PLCγ pathway. We previously reported that an increase in intracellular Ca^2+^ via IP_3_ receptor-sensitive intracellular Ca^2+^ storage, which is the downstream event of the TrkB/PLCγ activation, is essential for the BDNF-stimulated glutamate release in cortical neurons [18]. In the glutamate-release regulation by the BDNF/TrkB/PLCγ, an involvement of Src was also demonstrated [25]. As described below, p75 receptor negatively regulate neuronal function and it is possible that the expression balance of TrkB/p75 affects the vulnerability of brain neurons. Using a full-length knock-in (KI) Huntington’s Disease (HD) mouse model, Suelves et al. showed decreased striatal BDNF and TrkB levels, and a significant increase in p75 expression in the same brain region, in addition to motor coordination abnormalities in the animals. Importantly, normalization of p75 expression in KI-mutant mice by crossmating with p75 +/− mice delayed the onset of motor deficits. Furthermore, they found that an impaired TrkB-induced phosphorylation of PLCγ were improved in the KI mice by the normalization of striatal p75 levels although phosphorylation of ERK or Akt was not affected [26], implying TrkB/PLCγ-pathway specific action is involved in the altered behavior of the HD model animals. 

### 2.4. p75 Signaling Pathway

Although the p75 receptor is identified as a low-affinity receptor for all neurotrophins, the common receptor is recently recognized a high-affinity one for pro-neurotrophins [13]. Multimeric complexes p75 receptor and sortilin are involved in regulation of cell death promotion via biding with pro-neurotrophins. Asuni et al. have demonstrated an involvement of p75-mediated signaling in neuronal apoptosis caused by morphine withdrawal. They found that repeated cycles of spontaneous morphine withdrawal caused accumulated levels of p75 in hippocampal synapses while TrkB was decreased in mice. They also confirmed hippocampal apoptotic events including activation of c-Jun N-terminal kinase (JNK) and caspase-3. Importantly, it was revealed that the lack of one p75 allele (p75+/- mice) prevented the increased activation of JNK and caspase-3 [27]. In addition to the role in cell-death signaling, the p75 receptor negatively regulates synaptic function in the brain. Recent reports showed that the p75 receptor is involved in the age-dependent disruption of hippocampal homeostatic plasticity via the BDNF, ERK, and RhoA-ROCK2-LIMK1-cofilin signaling modulation [28]. Furthermore, it is possible that hippocampal structural and functional plasticity is also affected by p75 signaling. Sleep deprivation (SD) impaired hippocampal plasticity at both cellular and behavioral levels; however, mutant mice lacking the p75 expression were resistant to the deficits in neuronal plasticity. It was revealed that such a p75 deletion prevented the negative influence of SD on cAMP-CREB-BDNF, cAMP-PKA-LIMK1-cofilin, and RhoA-ROCK2 signaling in the hippocampus [29].

## 3. BDNF Signaling in AD

### 3.1. Downregulation of the BDNF in AD

AD is the most common neurodegenerative disease with progressive loss of neuronal cells, especially in cortical and hippocampal regions. Patients suffer from a significant decline in memory and cognitive function. AD is characterized by the accumulation of Aβ plaques and formation of neurofibrillary tangles, derived from hyperphosphorylated tau proteins. A number of studies examined levels of the BDNF protein in blood, CSF, and post-mortem brain tissue of AD patients [3,4,5]. Although there are several inconsistent reports, likely due to a variety of pathological stages of AD, different sample size, and condition of post-mortem brain tissue, the recent meta-analysis covering 98 articles supports the downregulation of the BDNF in blood, CSF, hippocampus, and cortex in AD patients [30]. The *BDNF* gene has several promoters governing nine upstream exons encoding 5′ untranslated regions (UTRs) that are spliced into one common downstream exon which contains the coding region and the 3′UTR, leading to nine different transcripts [31]. Garzon et al. showed that *BDNF* transcripts containing exon I, II, and IV were decreased in the prefrontal cortex of AD patients [3]. Furthermore, oligomeric Aβ reduced *BDNF* exon IV transcripts in human neuroblastoma cells in vitro [6]. An animal model of AD also exhibited a lower level of *BDNF* exon IV transcripts and the expression of the *BDNF* was negatively correlated with the level of oligomers. Overexpression of tau protein also caused the downregulation of the BDNF expression in human neuroblastoma cells and transgenic mice [32]. It was revealed that decreased BDNF levels in the Aβ overexpressed mice was rescued when the overexpressed mice were crossed with tau-knockout mice, suggesting tau pathology was also involved in Aβ-induced BDNF downregulation [32]. Expression of the *BDNF* promoters I, II, and IV are regulated by the transcriptional factor CREB of which transcriptional activity is dependent on the neuronal activity [31]. Considering that the AD brain exhibited decrease of phosphorylated CREB (activated form), reduced CREB activity is likely responsible for the BDNF downregulation.

### 3.2. Imbalance of proBDNF Transformation into Mature BDNF

First, the BDNF is translated as a precursor form and then processed by the intracellular/extracellular proteases into mature BDNF (mBDNF). Transformation of proBDNF into mBDNF is a critical step to regulate the mBDNF/TrkB signaling because proBDNF preferentially binds with p75/sortilin receptor but not with TrkB. In addition, contrarily to the positive influence of mBDNF on CNS neurons, proBDNF induces synaptic depression and apoptosis via p75/sortilin signaling [33,34]. In hippocampal tissue and CSF from AD patients, the expression of proBDNF, sortilin, and the ratio of proBDNF/BDNF were increased in comparison with healthy controls [9]. proBDNF in AD patients was highly modified with advanced glycation end products which prevents proteolytic cleavage by proteases. Administration of CSF from AD patients caused apoptosis in the primary culture of hippocampal neurons via p75/sortilin receptor-dependent manner. In addition to post-translational modification in the BDNF protein, the alternation of protease activity also influences the BDNF processing. Proteolytic cleavage of proBDNF into mBDNF was regulated by intracellular and extracellular proteases including mammalian Kexin-like proteases (Furin, PC 1/3/7), tissue plasminogen activator (tPA)/plasmin system (plasminogen, plasmin, tPA, plasminogen activator inihibitor-1 (PAI-1), and alpha (2)-antiplasmin), and matrix metallopeptidases 9 (MMP9) [33]. Interestingly, tPA/the plasmin system and MMP9 also cleave and degrade Aβ peptide. Therefore, activity of these proteases may impact both Aβ accumulation and mBDNF expression. In the neocortex of AD patients, the levels of plasminogen (pro-type of plasmin) and plasmin were lower than healthy controls [35]. Cai et al. reported that AD model mice showed a lower plasmin level in the hippocampus [36]. They also found that a treatment of spinosyn, a flavonoid isolated from Zizyphus jujuba var spinosa seeds, increased expression and activity of hippocampal plasmin and synaptic plasticity in AD model mice. The beneficial effect of spinosyn on synaptic plasticity was blocked by a plasmin inhibitor. Activity of tPA, an endogenous activator of plasmin, is also downregulated in the AD brain in accordance with an increased expression of neuroserpin, an endogenous inhibitor of tPA [37]. In addition, an upregulation of PAI-1, another endogenous inhibitor of tPA, was also reported in Aβ-treated primary neurons, the hippocampus of AD model mice and frontal cortex of AD patients [38]. Aβ peptide induced PAI-1 upregulation through the JNK/c-Jun pathways. The PAI-1 inhibitor resulted in improved BDNF maturation and cognitive function without affecting the burden level of amyloid. Further, cognitive stimulation given at a pre-plaque and pre-symptomatic phase protected from cognitive decline in accordance with an increase of PAI-1, decreased activity of tPA, and enhanced production of mBDNF in AD model mice. On the other hand, neuronal overexpression of MMP-9 in AD model mice resulted in increased soluble amyloid precursor protein α(sAPPα) levels, decreased Aβ oligomers, enhanced insulin signaling, and increased mBDNF levels, and prevented the cognitive deficits [39]. Hu et al. showed that PL402, rhamnoside derivative, suppressed the Aβ level via upregulation of MMP3/9 in cell models [40]. PL402 also attenuated Aβ pathology and cognitive defects in AD model mice with the consistent increase of MMP3/9. MMP9 also has a role in leukocyte migration from circulation into the brain under inflammatory conditions [41]. Indeed, knockout of MMP2 and MMP9 showed neuroprotective effects against inflammation in a murine model of experimental autoimmune encephalomyelitis [42,43]. Considering that neuroinflammation is one of the major pathologies of AD, it is required to carefully assess the possible impact of a protease enhancer on neuroinflammation. Collectively, enhancement of the BDNF and Aβ processing via proteases is a potential therapeutic strategy in AD, while it is required to take into account side effects.

### 3.3. Types of TrkB Receptors and AD

There are two type of TrkB receptors: full-length TrkB (TrkB-FL) and truncated TrkB (TrkB-TC); the latter lacks the C-terminal catalytic domain. TrkB-TC negatively regulates BDNF/TrkB signaling because heterodimer of TrkB-TC/TrkB-FL fail to stimulate the downstream signaling. TrkB-TC is generated through two pathways: transcription of *TrkB* gene (*NTRK2*) isoform and proteolytic processing of TrkB-FL. Santos et al. reported that Aβ treatment selectively increased mRNA levels for *TrkB-TC* isoform without affecting *TrkB-FL* mRNA levels in rat primary culture [44]. In addition, they found that Aβ induced calpain-dependent cleavage of TrkB-FL into TrkB-TC [44]. AD model mice also exhibited an age-dependent relative increase in cortical (but not hippocampal) TrkB-TC receptor levels compared with TrkB-FL [45]. Of note, overexpression of *TrkB-TC* in AD model mice exacerbated their spatial memory impairment while the overexpression of *TrkB-FL* alleviated it [45]. In the postmortem brain of AD patients, TrkB-TC was increased while TrkB-FL was decreased in prefrontal cortex regions [46]. The researchers also reported that TrkB-FL immunoreactivity was largely decreased in tangle-bearing neurons although increased TrkB-TC immunoreactivity in neurons and reactive astrocytes was confirmed. These studies suggest that an imbalance of TrkB-FL and TrkB-TC contributes to the disturbance of BDNF/TrkB signaling in AD.

### 3.4. The BDNF Polymorphism and AD

The *BDNF* gene carries more than 100 polymorphisms, which may influence BDNF signaling and the pathophysiology of AD [47]. The single nucleotide polymorphism rs6265, also known as the Val66Met polymorphism, is one of the most characterized polymorphism in relation to BDNF function and risk of brain disease [47]. This polymorphism leads to valine (Val) to methionine (Met) substitution at position 66 in the prodomain of the *BDNF*. In primary hippocampal neurons, the *BDNF_Met_* was inefficiently sorted into secretory granules, resulting in abnormal intracellular trafficking and failed localization in synapses [48]. As a result, activity-dependent secretion of the *BDNF_Met_* was lower than that of the *BDNF_val_*, which lead to an impairment of synaptic plasticity in the *BDNF_Met_* carrier [49]. Consistently, in human subjects, the met allele was associated with poorer episodic memory, with abnormal hippocampal activation monitored with fMRI [50]. The relationship between AD and rs6265 polymorphism was reported from several groups [51,52,53,54]. A preliminary study with a small sample size in Australian populations reported that Met carriers showed a significant and large decline in episodic memory and hippocampal volume but did not show significant changes in the rate of Aβ accumulation [51]. Another group also demonstrated that Met carriers exhibited a sharp decline in verbal learning/memory and speed/flexibility in a cohort consisting of 89% White, 8% Black, and 2% Hispanic populations [52]. In Japanese populations, a significant allelic association between rs6265 and AD was found in women, but not men [53]. On the other hand, study with Chinese Han populations reported no significant allelic association between rs6265 and AD [54]. These controversial results imply that the risk impact of rs6265 on AD is influenced by ethnic groups and sex.

### 3.5. Pathogenic Role of the BDNF in AD

Since the BDNF regulates neuronal survival, synaptic plasticity and memory, many research studies focused on the pathological role of BDNF downregulation in AD. In the primary culture of hippocampal neurons, a deprivation of NGF or BDNF induces Aβ production via upregulation of amyloidogenic pathway components, APP, presenilin-1 [55]. It was also reported that BDNF neutralization or knockout leads to increased pro-inflammatory cytokines and activates the JAK2/STAT3/C/EBPβ pathway, resulting in the upregulation of asparagine endopeptidase-mediated APP and Tau cleavage, generating Aβ and neurotoxic Tau N368 [56]. Rohe et al. reported that the BDNF upregulated SOLRA expressions through the ERK signaling pathway and reduced amyloidogenic processing in primary cortical neurons [7]. BDNF treatment also dephosphorylated tau protein at S202, T205, AT180, and S262 sites in neuronally differentiated P19 mouse embryonic carcinoma cells through the PI3K/Akt signaling pathway [8]. Although in vitro studies suggest an involvement of the BDNF function in amyloid processing and tau phosphorylation, there are conflicting reports on the role of the BDNF in Aβ and tau pathology in AD mice models. Braun et al. performed conditional depletion of hippocampal BDNF in adult AD model mice [57]. Interestingly, the BDNF depletion reduced noradrenergic neurons in the locus coeruleus (LC) and noradrenergic projection in the hippocampus, frontal cortex, and molecular layer of the cerebellum. In addition, the number of microglia was decreased while Aβ plaque was increased in the cortex of the BDNF-depleted AD model mice. This study suggested that loss of hippocampal BDNF affects the cortex via decreasing LC projection and leads to increased Aβ plaque due to reduced microglial phagocytosis. The small molecule TrkB agonist, 7,8-DHF, decreased cortical Aβ plaque deposition and protected cortical neurons against reduced dendritic arbor complexity in AD model mice [58]. On the other hand, Castello et al. demonstrated that both Aβ and tau pathology in AD model mice were not influenced when the mice were crossed with heterozygous *BDNF* knockout (+/−) mice [59]. Heterozygous knockout of *TrkB* in the AD model mice also did not show any change in Aβ pathology although memory decline was exacerbated [60]. The *BDNF* overexpression of astrocyte in AD model mice rescued the BDNF signaling activity associated with an improvement of dendritic spine density and morphology, synaptic plasticity, and behavioral memory/cognitive performance without any improvement of Aβ plaque deposition [61]. The inconsistency on the impact of BDNF signaling on Aβ and tau pathology may be due to different experimental strategies to achieve BDNF downregulation or upregulation. Further studies are required to clarify the BDNF function in AD pathology.

### 3.6. The BDNF, Dysregulation of the Cholinergic System, and Dementia in Down Syndrome

Decreased function of the central cholinergic nervous system, including lack of acetylcholine neurotransmitter, is also recognized as one of the key mechanisms underlying the cognitive dysfunction in AD patients [62]. It was demonstrated that typical phenotypes of AD progression including loss of cholinergic neurons and reduced activity of choline acetyltransferase in the nucleus basalis of Meynert are closely related to deficits in memory and the cognitive function of AD because the cholinergic neurons in the region innervate to the hippocampus and neocortex (see the review by Cheng et al. 2021) [63]. Importantly, basal forebrain cholinergic neurons (BFCNs) depend their synaptic function and cell survival on the BDNF, which is retrogradely released from BFCN targets. Interestingly, using an in vitro aging model with rat BFCNs, Shekari and Fahnestock found impaired axonal transport of the BDNF protein [64]. They also observed that proNGF was transported in BFCNs and its activity was diminished during in vitro age progression. The aged BFCNs displayed decreased expression of both TrkA and TrkB, although levels of p75 were not changed during in vitro aging, implying the vulnerability of BFCNs in AD may be due to the downregulation of neurotrophin transport. It was reported that Rh2, a rare ginsenoside, has neuroprotective effects including increased activity of choline acetyl transferase (ChAT) against scopolamine-induced memory deficits in mice [65]. Furthermore, treatment with Rh2 induced the significant upregulation of activated ERK and CREB, and expression of the BDNF in the hippocampus of the memory-deficit model. Recently, the effect of dl-3-n-butylphthalide (NBP) on hippocampal expression levels of ChAT, acetylcholinesterase (AChE), vesicular acetylcholine transporter (VAChT), and the BDNF was examined. A rat model of vascular dementia established by bilateral common carotid artery ligation exhibited decreased spatial learning and memory evaluated with a Morris water maze test, although administration of NBP reversed the decreased learning and memory function [66]. NBP treatment also increased ChAT, AChE, VAChT, and BDNF expressions, suggesting that central cholinergic dysfunction is involved in vascular dementia pathogenesis and NBP is effective to protect the cholinergic system via activating the BDNF signaling [66]. Interestingly, the influence of focused ultrasound (FUS)-mediated blood–brain barrier opening in the cholinergic degeneration rat model was reported [67]. Significant cholinergic degeneration, decreased hippocampal neurogenesis, and deficits in spatial memory function after an administration of 192 IgG-saporin (a cholinergic immunotoxin), were all rescued by FUS-mediated brain–blood barrier opening, with a marked upregulation of the BDNF.

Critical involvement of cholinergic dysregulation in the AD pathogenesis is likely, judging from molecular and behavioral phenotypes of Down syndrome (DS). DS, a genetic developmental disorder due to trisomy 21, exhibits dementia by the third or fourth decade of life such as in early-onset AD. As expected, significant atrophy of BFCNs in DS was also shown [68]. Previously, using a DS model animal, Ts65Dn mouse, which is recognized as a useful animal model for DS and AD, significant deficits in the Morris water maze performance, cholinergic neuronal degeneration of ChAT-positive neurons, and increased APP protein levels in the hippocampus were demonstrated [69]. It was also shown that the Ts65Dn mouse exhibited decreased neurogenesis in the hippocampus [70]. Interestingly, additional choline (approximately 4.5 times than control) supplementing the maternal diet improved the performance of the adult Ts65Dn offspring in a radial arm water maze task with partial normalization of the decreased hippocampal neurogenesis [70]. Although no therapies for intellectual disability in DS are established, the potential effect of 7,8-DHF on the behavior of the Ts65Dn model is very interesting [71] as 7,8-DHF, a small molecule agonist for TrkB, was demonstrated to exert beneficial effects in various brain disease models including AD animals. Giacomini et al. found that Ts65Dn mice treated with 7,8-DHF during postnatal days P3-15 did not display any learning and memory improvement (Morris Water Maze) at 1 month later. Ts65Dn mice receiving 7,8-DHF for about 40 days starting from 4 months of age also did not show any improvement in learning and memory. The same group previously reported that the early treatment with 7,8-DHF (for 40 days, from P3 to 45) restored deficits in learning and memory and decreased hippocampal neurogenesis in Ts65Dn mice, suggesting that a timing of treatment with the flavonoid, 7,8-DHF, is very critical [72]. Considering that an improvement of cholinergic degeneration and resultant decreased hippocampal neurogenesis after the disease onset is very difficult, available combined application (e.g., FUS and 7,8-DHF) using the DS or the other early-onset AD models during development may be beneficial in future studies. 

## 4. Therapeutic Strategy Targeting Neurotrophin Signaling

### 4.1. Direct Delivery of the BDNF

Considering the protective effect of the BDNF in AD pathology, the BDNF and its downstream signaling seem to be an attractive target for AD therapy. Treatment with recombinant BDNF protein or delivery of the *BDNF* gene using lentiviral vector were reported in animal studies. Lentiviral *BDNF* gene delivery into the entorhinal cortices at a young age (2 months old) prevented cell loss and improved the downregulation of synaptic proteins in the hippocampus and hippocampal-dependent contextual fear conditioning without affecting the deposit level of amyloid in AD model mice [73]. Furthermore, lentiviral *BDNF* gene delivery after disease onset (6 months old) also reversed synapse loss, partially normalized aberrant gene expression, improved cell signaling, and restored learning and memory in AD model mice [74]. In aged rats, an infusion of the BDNF protein into the medial entorhinal cortex for 4 weeks improved the age-dependent decline of spatial learning and memory [74]. Transplantation of stem cells expressing high levels of BDNF is another strategy to deliver the BDNF into the CNS. Neural stem cell (NSC) transplantation in aged AD model mice rescued spatial learning and memory deficits and reduced hippocampal synaptic density without affecting Aβ plaque and neurofibrillary tangle pathology [75]. Interestingly, a depletion of the BDNF from NSCs diminished beneficial effects to improve cognition or restore hippocampal synaptic density, suggesting an essential role of NSC-derived, BDNF in therapeutic effects. These studies provide proof of concept for BDNF-targeting AD therapy; however, direct infusion, lentiviral gene transfer, and transplantation are not clinically feasible due to their high-invasiveness and the limited diffusion of the BDNF protein/viral/cell from injected sites. Intranasal delivery is not invasive and can bypass the BBB and deliver the drug into the CNS. In this route, the BDNF reached the cortex and hippocampus at 0.1–1 nM in mice [76]. Recently, Braschi et al. showed that intranasal delivery of the BDNF after the onset of cognitive and anatomical deficits (6 months old) rescued memory deficits and reduced the number of reactive microglia without affecting Aβ burden, tau hyperphosphorylation, and cholinergic deficit in AD11 mice for a model of NGF deprivation-induced neurodegeneration [77]. This study suggests the possible usefulness of intranasal delivery; however, it should be investigated whether the BDNF can diffuse into the cortex and hippocampus at an effective dose in a large human brain.

### 4.2. A Flavonoid Agonist for TrkB Activation—Preclinical Study

Systemic treatment with small compounds which can pass through the BBB and induce an upregulation of the BDNF expression may offer a much better therapeutic strategy for AD. Hexadecanamide (Hex) is an endogenous ligand of peroxisome proliferator-activated receptorα which induces BDNF expression in hippocampal neurons [78]. Oral administration of Hex increased the concentration of Hex in the hippocampus and upregulated the BDNF and its downstream plasticity-associated molecules, resulting in improved synaptic plasticity and memory/learning in AD model mice [78]. Flavonoids are a large family of dietary polyphenol compounds, are found almost ubiquitously in plants, and some of them have ability to activate BDNF signaling [79]. As shown above, 7,8-DHF, a derivate of flavone, was identified as a selective small-molecular TrkB agonist that mimics the physiological actions of the BDNF [10]. Oral administration of 7,8-DHF from a young age (2 months old) can activate TrkB receptors in the brain, as evidenced by an increase in phosphorylated levels of TrkB. The flavone administration prevented Aβ deposition, the loss of hippocampal synapses, synaptic dysfunction, and spatial memory deficits in AD model mice [80]. Intraperitoneal injection of 7,8-DHF into CaM/Tet-D_TA_ mice, an inducible model of severe neuronal loss in the hippocampus and cortex, also rescued poorer spatial memory and reversed a decreased density of thin synaptic spines in the CA1 region (no affecting mushroom or stubby spines) [81]. Recently, in order to improve oral bioavailability and pharmacokinetic profiles, R13, a prodrug of 7,8-DHF, was developed [82]. R13 shows favorable pharmacokinetic profiles (PK) in in vitro absorption, distribution, metabolism, and excretion assays, combined with in vivo PK studies. Oral administration of R13 in AD model mice activated TrkB signaling and prevented Aβ deposition, the loss of hippocampal synapses, and memory deficits in a dose-dependent manner [82]. These studies suggest the potential usefulness of 7,8-DHF and its derivates for AD therapy; therefore, the safety and tolerability for humans would be needed for future clinical use. Besides 7,8-DHF and other flavonoids also show an indirect but significant effect on BDNF signaling. Labban et al. examined the effect of melatonin and resveratrol— the former is a pineal product and the latter a polyphenolic compound extracted mainly from grapes and other fruits—on AD model mice [83]. Both compounds restored memory deficits and decreased the BDNF expression in the prefrontal cortex of AD model mice. Melatonin also influences CREB expression. Yan et al. reported the beneficial effect of flavonoids extracted from okra fruit (FOF), which is a tropical vegetable that has been used as a natural drug in east and west Africa for many centuries [84]. Intragastric infusion of FOF for 4 weeks in acute model of AD (with intracerebroventricular injection of Aβ1–42) upregulated BDNF expression levels and activated CREB/ERK and PI3K/Akt/GSK-3β signaling pathways and was accompanied by an improvement in cognitive deficits. Astilbin (AST) is isolated from the rhizome of Smilax china L and widely used in traditional Chinese medical treatment [85]. Intraperitoneal administration of AST for 8 weeks in AD model mice showed beneficial effects on lessening learning and memory deficits. The AST treatment reduced plaque burden and Aβ levels, and enhanced BDNF expression, CREB activation, and PI3K/GSK-3β signaling. Quercetin, a natural polyphenol, is abundantly found in vegetables and fruits. Oral administration of quercetin for 1 month in a rat model of AD induced by Aβ injection promoted learning and memory performance and expression of the *BDNF*, *NGF*, *CREB*, and *EGR-1* genes [86]. In addition, quercetin enhanced adult neurogenesis in hippocampal dentate gyrus. Regarding the relationship between BDNF signaling and neurogenesis, please refer to our previous review [14]. Collectively, some kinds of flavonoids can induce activation of BDNF signaling even if they are administrated systemically. Due to the wide spectrum of pharmacological efficacies, detailed molecular mechanism underlying the BDNF upregulation stimulated by these flavonoids are still unclear; therefore, determination of the target of these flavonoids is beneficial for drug development in future studies.

### 4.3. Potential Benefit of Flavonoids in Improvement of Cognitive Decline—Clinical Study

Several epidemiological studies suggest the beneficial effect of flavonoids on cognitive function in humans [87]. High dietary consumption of vegetables is associated with a slower rate of cognitive decline with older age [88]. Intake of flavonoid-rich wine, tea, and chocolate was associated with better performance in cognitive abilities in the elderly [89]. Moreover, a recent study has demonstrated that individuals with long-term dietary intake of flavonols, anthocyanins, and flavonoid polymers had a lower risk of AD-related dementia [90]. As a therapeutical approach, EGb761, which is a standardized *Ginkgo biloba* extract (GbE) containing flavonoids and terpenoids, was intensively examined and discussed whether it alleviates symptoms associated with cognitive disorders. In a meta-analysis to assess the efficacy and safety of EGb761 for dementia and/or cognitive decline, Birks et al. demonstrated that the four independent trials showed inconsistent results: three of them found no difference between EGb761 and a placebo, while one trial indicated a large treatment effect in EGb761-treated group [91]. A recent meta-analysis reported that GbE has potentially beneficial effects over a placebo on cognitive performance in dementia after being treated with a dose greater than 200 mg/day for at least 5 months; however, they also pointed a lower quality of evidence [92]. Therefore, clinical evidence of the use of flavonoids for AD therapy is still insufficient; further rigorously designed, multicenter-based, large-scale RCTs are warranted.

## 5. Conclusions

**Figure 2:**
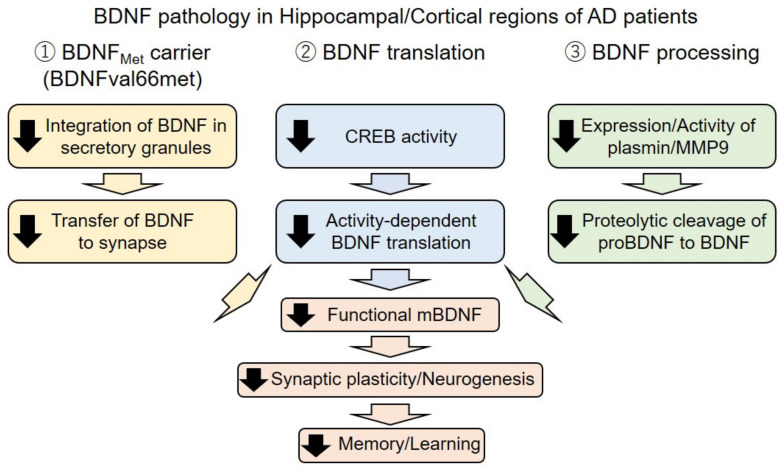
Schematic illustration of dysregulation of the BDNF in Alzheimer’s disease (AD). In the AD brain, mature BDNF (mBDNF) expression is affected by at least three distinct mechanisms, including gene polymorphism (rs6265, BDNF val66met), mRNA translation, and BDNF processing. Through these mechanisms, a reduction of functional mBDNF, impairment of synaptic plasticity/neurogenesis, and then significant decline in memory and learning functions are caused. Black arrow: downregulation.

In this review, we introduced an essential role of BDNF signaling in neural functions from cellular to behavior levels, especially focusing on learning and memory functions. We also reviewed the current evidence of BDNF dysfunction in AD at gene, mRNA, and protein level and discussed possible involvement in the pathophysiology of AD and potential usefulness in BDNF-targeting therapy (Figure 2). Because AD is a progressive and irreversible disease, suppressing the progress of the pathology to prevent the onset and worsening of symptoms is a feasible strategy. Currently, drug development for AD treatment mainly targets Aβ or tau, such as a BACE inhibitor or anti-Aβ and/or anti-tau antibodies, in order to suppress the production or remove of pathogens. On the other hand, BDNF-targeting therapy provides a different strategy, which aims to provide trophic support to degenerating neurons and enhance synapse plasticity against AD-related pathology. In this sense, the BDNF is a promising target for AD therapy because an additive/synergistic effect in case of combinational use with the Aβ/tau-targeting therapy is also expected. Unfortunately, treatment with an Aβ-targeting drug failed to show strong evidence of its efficacy in clinical trials at this moment, causing a great debate to review the amyloid cascade hypothesis in the AD pathogenesis. However, the occurrence of familial AD with mutation in APP and presenilin (a component of γ-secretase which cleaves APP into Aβ) strongly supported the pathogenic role of Aβ in AD. These discrepancies may indicate that the pathogenic role of Aβ is limited at the onset of the disease to induce, for example, tau pathology and/or BDNF downregulation, and is not directly responsible for the progress of neurodegeneration. This hypothesis is consistent with an idea that hallmarks of Aβ accumulation is progressed more than a decade before the emergence of synaptic loss, neural injury, and symptomatic memory loss [93]. Therefore, preventive therapy started in the presymptomatic stage would be applicable. Judging from animal studies introduced here, the BDNF-targeting therapy of small compounds (e.g., flavonoids) also show significant efficacy when administrated from the presymptomatic stage. Therefore, in the future, presymptomatic treatment with combinational use of Aβ/tau- and BDNF-targeting therapy should be explored for therapeutic intervention in AD.
